# Do asymptomatic patients have normal function after percutaneous fixation of the posterior pelvic ring? A case-control pilot study

**DOI:** 10.1186/s13018-015-0190-z

**Published:** 2015-05-16

**Authors:** Pooria Salari, Lisa K Cannada, Berton R Moed

**Affiliations:** Department of Orthopaedic Surgery, Saint Louis University School of Medicine, 3635 Vista Avenue, 7th Floor Desloge Towers, St. Louis, MO 63110 USA

**Keywords:** Pelvic fracture outcome, Percutaneous iliosacral screws, Type C pelvic ring injuries

## Abstract

**Background:**

Following treatment of a posterior pelvic disruption, residual deformity or associated injuries can adversely affect functional recovery. No study has been performed on gait and functional outcome after closed reduction and percutaneous screw fixation (CRPSF) of posterior pelvic disruption in clinically asymptomatic patients. The purpose of this study was to determine if gait and functional outcome are different from normal in asymptomatic patients with a posterior pelvic injury after CRPSF, serving as a pilot study in this regard.

**Methods:**

Six asymptomatic patients with no grossly evident gait abnormality, treated by CRPSF for a posterior pelvic disruption, were included in the study (SG). A control group (CG) of six healthy volunteers was created. All participants completed the 12-Item Short Form Health Survey version 2 (SF-12v2), the Majeed Pelvic Score (MPS), and the Iowa Pelvic Score (IPS). In addition, the participants’ gait was analyzed.

**Results:**

Pelvic drop was significantly smaller on the uninjured side in the SG when compared to the injured side in the SG. There was no significant difference between the injured and uninjured side for other gait parameters within the SG. Knee angle at initial contact was significantly greater on the injured side when compared to the CG. The SG scored statistically worse than the CG on the Physical Component Summary part of the SF-12v2. However, when evaluated by age group using national mean scores, the SG differences were minimal. All six patients in our study scored “excellent” on both MPS and IPS.

**Conclusions:**

Despite having subclinical alterations in gait, asymptomatic pelvic ring injured patients show minimal, if any, evidence of impaired functional outcome following successful reduction of a posterior pelvic disruption treated by CRPSF.

## Background

Pelvic fractures account for 1%–3% of all skeletal fractures and 2% of orthopedic hospital admissions [1,2]. These fractures comprise a broad spectrum of injuries, from low-energy slip-and-fall fractures in osteoporotic patients to high-energy disruptions caused by motor vehicle accidents. Following treatment of these pelvic injuries, residual deformity or associated injuries are felt to adversely affect functional recovery [3-9]. Type C injuries to the posterior aspect of the pelvic ring with sacroiliac joint dislocation or fracture/dislocation and displaced sacral fracture are associated with higher morbidity and mortality [6,10,11]. Conventional wisdom is that in general these patients do not do well. Long-term medical and socioeconomic implications of pelvic fractures are well documented; these include mental health problems, chronic pain, pelvic obliquity, leg length or rotational discrepancy, sexual and urological dysfunction, and long-term unemployment [9,12-16].

Objective evaluation of gait and specific outcomes of type C pelvic ring fractures has been very sparsely studied. To our knowledge, no study has been performed on gait pattern and functional outcome after closed reduction and percutaneous screw fixation (CRPSF) of posterior pelvic ring disruption in clinically asymptomatic patients.

The purpose of this study was to take a closer look at those who are believed to be doing well by determining if gait parameters and functional outcome are different from normal in asymptomatic patients with a displaced posterior pelvic ring injury after CRPSF. We hypothesized that there would be significant gait disturbance and impaired functional outcome in patients with a posterior pelvic ring disruption, as compared to an uninjured population, despite being clinically asymptomatic.

## Methods

Between January 2008 and May 2012, 72 patients underwent CRPSF using FDA-approved devices for a type C pelvic ring injury [11] having a sacral fracture, sacroiliac dislocation, or sacroiliac fracture/dislocation at our institution. All radiographic studies, operative reports, and the most recent clinic follow-up visit notes were reviewed in this Saint Louis University institutional review board-approved pilot study. This was performed in accordance with the Declaration of Helsinki and was approved by our ethics committee (Protocol Number: 22378). To be considered for the study, patients must have been at least 1 year postoperative and considered themselves to be completely asymptomatic. They must also have met the following inclusion criteria: 1) age between 18 and 65 years; 2) absence of other ipsilateral or contralateral lower extremity fracture and any associated neurologic injury; 3) availability for review of the preoperative radiographs and computerized tomography (CT) scans, immediate postoperative radiographs, and follow-up radiographs; 4) an excellent reduction after CRPSF (defined as less than 5 mm of displacement [17]); 5) evidence of union on the latest follow-up radiographs; 6) maintained reduction of the pelvic ring; 7) absence of leg length discrepancy on radiographic and clinical exam; 8) no subjective complaints of limp; 9) no regular use of pain medication; and 10) physical ability to walk on a treadmill. Specific exclusion criteria consisted of the following: 1) failure to meet any of the inclusion criteria, 2) bilateral posterior ring injury, 3) associated acetabular fracture, and 4) new injury or disability with onset after the index pelvic injury.

Twelve patients met all of the above criteria, and six of these patients agreed to participate in this study (four patients refused because of transportation issue, and two patients demanded higher amount of compensation than what was approved by our facility’s institutional review board). Each patient received a $50 gift card for his/her participation in this study. The study group (SG) consisted of five males and one female. Average age at the time of the injury was 45 years (range, 21 to 62 years); average body mass index (BMI) was 27.8 (range, 17.2 to 38); average time of follow-up was 13 months (range, 12 to 15 months). Three patients had an injury on the right side and three on the left side. At the time of injury, all patients were employed outside the home. In none of these patients was the index pelvic ring disruption a work-related injury. The mechanism of injury was a motor vehicle crash in four and a fall from a height in two patients. Using the OTA/AO classification [11], all fractures were 61-C1, being either a sacroiliac fracture/dislocation (two patients) or a sacral fracture (four patients). One patient had associated facial injuries, and two had upper extremity fractures, including one of the contralateral distal radius and one of the ipsilateral clavicle fracture. Other injuries, including liver and splenic laceration, required no surgical treatment. Two subjects were treated for high blood pressure, and one had diabetes.

Each of these six patients had undergone closed reduction and percutaneous screw fixation of the pelvic ring injury by one of two pelvic surgery specialists at our institution. Closed reduction was obtained in all cases by a variety of means including application of skeletal traction and/or application of an anterior external fixation device and/or initial internal fixation of the pubic symphysis. Intraoperative fluoroscopic anteroposterior, inlet and outlet pelvis images were used to confirm adequacy of reduction and to direct screw placement. Operative stabilization of the posterior ring after reduction was performed using 6.5-, 7.0-, or 7.3-mm cannulated screws. Postoperatively, each patient was instructed to maintain nonweight bearing for 3 months on the injured side, during which time motion at the hip, knee, and ankle was encouraged. Each patient had been instructed to follow up at 2, 6, and 12 weeks, 6 and 12 months, and then yearly. Physical examinations had been performed, and plain radiographs were obtained at each visit with the findings documented. In general, patients were advanced to full weight bearing at 3 months postoperatively and began a physical therapy course that included generalized lower extremity muscle strengthening and ambulation training.

In addition, a control group (CG) was recruited from our institution for comparison, consisting of six healthy individuals with no history of pelvic or lower extremity injury or other pathology (mean age, 29 years (range, 26 to 36 years); mean BMI, 24.5 (range, 21 to 30.4)). These volunteers had no complaints of pain and had no orthopedic problems at the time of testing.

**Figure 1:**
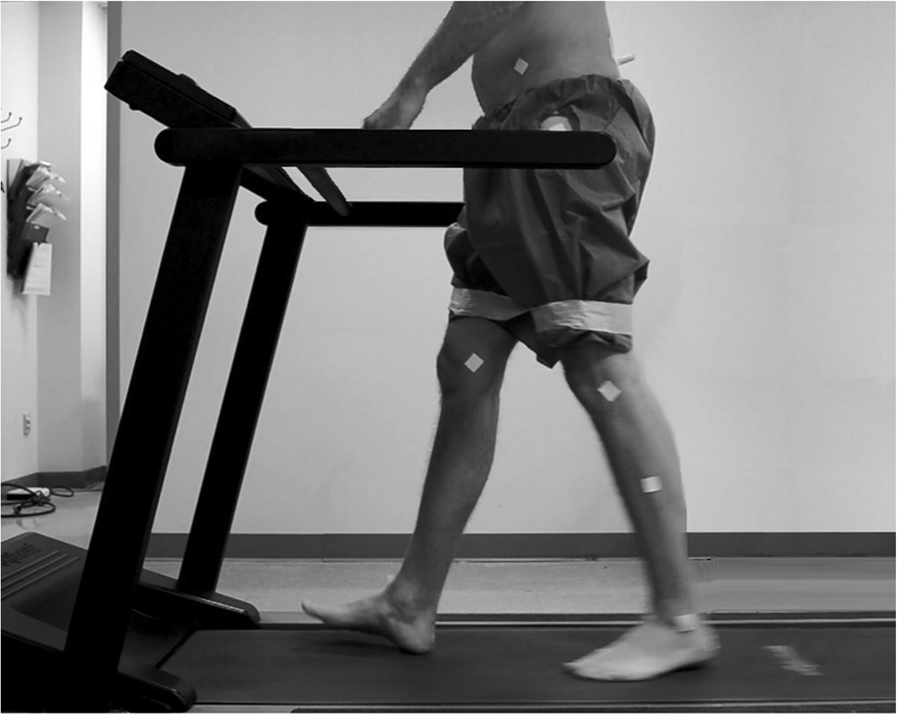
This lateral view of a study subject during gait evaluation shows several reflective markers placed on the pelvis, thighs, legs, and feet for motion analysis.

After obtaining consent at the time of gait evaluation, each subject was asked to wear single-use paper shorts and top for adequate exposure of bony landmarks. Multiple reflective surface markers were placed at the same anatomic locations (on the trunk, thighs, legs, and feet) of each subject (Figure 1). With comfortable walking speed being 2–3 mph, to increase measurement accuracy, 2 mph walking speed was chosen for this study [18]. Therefore, all participants walked barefoot on a treadmill at the pace of 2 mph. Each subject’s gait was video recorded from right, left, front, and back. Video data from a Nikon D5200 HD camera was then imported into a computer-based gait analysis software (Dartfish™ software, Fribourg, Switzerland). For each data point, multiple angles were measured and then averaged. These data were then analyzed for eight specific recognized gait variables. The variables included the following: maximum foot dorsiflexion during stance (DFDS), maximum knee flexion during stance (KFDS), knee angle at initial contact (KAIC), pelvic tilt at midstance (PTMS), pelvic tilt at initial contact (PTIC), pelvic tilt at heel off (PTHO), stride length (SL), and maximum pelvic drop (PD) [19].

At the time of gait evaluation, each subject completed three functional outcome questionnaires: the 12-Item Short Form Health Survey version 2 (SF-12v2) and two pelvic-specific outcome questionnaires, the Majeed Pelvic Score (MPS) and the Iowa Pelvic Score (IPS). The SF-12v2 is a validated 12-item self-administered health status questionnaire intended to assess self-perception of physical and psychological well-being [20]. The MPS comprises seven items: pain, work, sitting, sexual intercourse, standing, gait unaided, and walking distance [21,22]. The suggested cutoffs for excellent, good, fair, and poor results in those working before the injury are >85, 84–70, 69–55, and <55, respectively; for those not working before the injury, the suggested cutoffs are >70, 69–55, 54–45, and <45, respectively [22]. The IPS comprises six items: activities of daily life, work history, pain, limping, visual pain line, and cosmesis [23]. The grading scale is as follows: excellent, 100–85; good, 84–70; fair, 69–55; and poor, <55 [14,16]. While neither of these pelvic scores has been completely validated, they are in common use for the evaluation of patients with pelvic injuries [14,24].

The statistics were calculated using the SPSS 19.0 software (Chicago, IL). The Mann-Whitney *U* test was used to determine whether significant differences existed between the age, BMI, and gait of the patients in the SG and the CG. A Wilcoxon test was used to compare the injured side with the uninjured side within the SG. A *p* value <0.05 was considered to be significant.

## Results

There was no statistically significant difference between the SG and CG for age (*p* = 0.093) and BMI (*p* = 0.24). Analysis of the kinematic data revealed a statistically significantly greater KAIC on the injured side in the study group when compared to the right and left sides in the CG (*p* < 0.018). PD was significantly smaller on the uninjured side in the SG when compared to the injured side in the SG (*p* = 0.028) and to either side in the CG (*p* < 0.038). No statistically significant differences were found between the SG and CG with regard to the other parameters: stride length, dorsiflexion during stance, knee flexion during stance, pelvic tilt at midstance, pelvic tilt at initial contact, and pelvic tilt at heel off (Table 1). There were no significant gait parameter differences between the right and left sides within the CG.

**Table 1:** **Gait parameters of the study and control groups measured using the Dartfish™ software**

	**DFDS (degrees)**	**KFDS (degrees)**	KAIC* (degrees)	**PTMS (degrees)**	**PTIC (degrees)**	**PTHO (degrees)**	**PD** ^**†**^ **(degrees)**	**SL (m)**
Injured	8.7 ± 0.36	35.6 ± 6.3	11.1 ± 3.0	12.0 ± 7.4	10.7 ± 5.7	14.7 ± 7	3.7 ± 2.4	1.0 ± 0.2
Uninjured	9.4 ± 1.7	38.1 ± 3.3	8.9 ± 3.7	11.7 ± 4.8	9.8 ± 4.2	13.5 ± 4	0.17 ± 0.4	1.0 ± 0.2
Right side (control group)	9.8 ± 1.9	38.2 ± 3.6	4.9 ± 1.4	7.8 ± 3.9	7.0 ± 4.0	9.3 ± 4.5	2.7 ± 1.4	1.2 ± 0.06
Left side (control group)	10.1 ± 2	38.1 ± 4.6	5 ± 2.7	6.8 ± 5	6.4 ± 4.8	8.4 ± 4.9	2.2 ± 1.6	1.2 ± 0.05

*DFDS* maximum foot dorsiflexion during stance, *KFDS* maximum knee flexion during stance, *KAIC* knee angle at initial contact, *PTMS* pelvic tilt at midstance, *PTIC* pelvic tilt at initial contact, *PTHO* pelvic tilt at heel off, *PD* pelvic drop, *SL* stride length.

*KAIC greater on the injured side in the SG when compared to the right and left sides in the CG (*p* < 0.018).

^†^PD smaller on the uninjured side when compared to the injured side in the SG (*p* = 0.028) and to either side in the CG (*p* < 0.038).

The average SF-12v2 scores for Physical Component Summary (PCS) were 53.1 and 57.2 for the SG and CG, respectively, with the SG being significantly worse (*p* = 0.038). The Mental Component Summary (MCS) scores were 53 for the SG and 54.8 for the CG (*p* = 0.146) (Table 2). However, when the scores for the SG were compared to the appropriate-for-age-group national mean scores, the PCS score differences as well as those for the MCS scores were minimally, if at all, clinically important (Table 3). In addition, all six patients in our study scored “excellent” on both MPS and IPS, and there was no significant difference in the MPS questionnaire score between the SG and CG. However, the SG scored significantly lower than the CG on IPS (*p* = 0.022) (Table 4).

**Table 2:** **SF-12v2 questionnaire scores for the study group versus the control group**

		**Study group**		**Control group**	
		**Mean**	**SD**	**Mean**	**SD**
SF-12v2	PCS*	53.1	4.9	57.2	0.13
	MCS^†^	53	4.8	54.8	2.6

*p = 0.038, ^†^ *p* = 0.146.

**Table 3:** **SF-12v2 questionnaire scores for the study group compared to national mean scores for the age group**

**Subject number**	**Age group (years)**	**Subject PCS**	**National mean PCS score for age group** ^**a**^	**Subject MCS**	**National mean MCS score for age group** ^**a**^
1	18–24	50.9	53	46.6	46
2	25–34	52.8	53.3	49.6	48.9
3	35–44	56.6	52	60.8	48.8
4	35–44	44.5	52	54	48.8
5	45–54	56.9	49.4	54.7	49.9
6	65–74	57.3	43	52.8	51.6

^a^See [33].

**Table 4:** **Iowa and Majeed outcome scores for the study group versus the control group**

**Outcome score**	**Study group**		**Control group**	
	**Mean**	**SD**	**Mean**	**SD**
Iowa*	95	5.5	100	0
Majeed^†^	98	2.6	100	0

*p = 0.022, ^†^ *p* = 0.366.

## Discussion

Displaced posterior pelvic fractures are associated with significant complications and poor outcomes [3,13,14,16,21,25-27]. Numerous investigators have found that displacement through the weight-bearing arch of the pelvis can lead to long-term medical and socioeconomic problems. These include mental health problems, chronic pain, pelvic obliquity, leg length or rotational discrepancy, gait abnormalities, sexual and urological dysfunction, and long-term unemployment [13,14,20].

As the use of outcome instruments to report functional outcomes has become standard in the surgical literature [28,29], more reports have been published on functional outcomes after pelvic fractures using generic instruments [14]. In addition, several pelvic-specific outcome instruments have been used in the past two decades, with Majeed’s [21,22] and the Iowa [23] outcome instruments being the most widely used [14]. By using these outcome instruments, several authors have reported functional outcomes to be associated with many factors, including age, Injury Severity Score, type of fracture, location of fracture, residual posterior displacement, force vectors, treatment methods, open fracture, work-related injury, lower extremity fracture, urological injury, impotence, psychological problems, and neurological injury [13,29,30].

All the above-mentioned questionnaires have a gait component; however, the extent of gait disturbance is relatively unknown because these reports are generally based on subjective evaluation [19]. Significant abnormalities in gait parameters have been shown to occur after treatment of lower extremity fractures [31], pelvic fracture after open reduction and internal fixation [32], and acetabular fractures [19]. However, no study has been done to objectively evaluate a patient’s gait after CRPSF for sacral fracture and sacroiliac dislocation. To the best of our knowledge, this study is the first to assess functional outcomes and gait parameters after CRPSF for posterior pelvic ring disruption in otherwise normal patients and represents our experience in a small group of patients after CRPSF of sacral fractures and sacroiliac disruption.

One of the major limitations of this study is the small number of patients. This factor may have reduced the authors’ ability to discern differences between the patient’s affected and unaffected limbs and as compared to the control group. The small number of patients also precluded any age-specific statistical comparison, allowing only a descriptive analysis, of SF-12v2 PCS and MCS scores between the study group and those values considered to be normal for the United States population [33]. Nonetheless, the number of patients is satisfactory to serve as a pilot for future investigations, which was the object of this study. In addition, our study group patients were not specifically matched with the control group individuals for the variables age and BMI. However, in the absence of a statistically significant difference between the two groups for these two variables, differences in gait parameters are less likely to be the result of age or BMI differences. In addition, 1-year follow-up may not be indicative of longer-term result. However, these patients were completely asymptomatic and therefore, had most likely attained their full rehabilitation potential. Lastly, we used Dartfish™ gait analysis software which provides less information about gait kinematics than more complex three-dimensional gait analysis techniques. However, for the purpose of this study and in the presence of a control group, the data provided by this method was thought to be adequate for a pilot study.

Analysis of the kinematic data showed an alteration in two gait parameters. Pelvic drop was significantly smaller on the uninjured side in the study group when compared to the injured side in the SG, and both sides in the control group. KAIC was significantly more on the injured side when compared to the right and left sides in the CG. While the importance of these findings is unknown, they are unlikely to be clinically relevant in the absence of subjective limp. In a similar study of acetabulum fracture patients, when muscle strength was measured objectively and related to observed gait, an overall muscle strength deficit of 27% was observed [26]. This deficit was encountered despite a rehabilitation regimen and was considered by the authors to be a direct result of the surgical dissection. Since no surgical dissection is involved in the percutaneous insertion of iliosacral screws, one possible explanation for the somewhat abnormal gait parameters in this group of patients is asymmetric strength of the muscles involved in the gait cycle caused by the injury.

Although the MPS and IPS questionnaires may be susceptible to problems of content validity, internal consistency, and reproducibility, in the absence of a fully validated pelvic-specific outcome instrument, they have been widely used and accepted in literature [14]. All six patients in our study scored “excellent” on both MPS and IPS [21-23]. However, the raw scores of the SG were significantly lower than those of the CG on the Iowa Pelvic Score. Furthermore, the SG patients scored significantly worse than the CG on PCS of SF-12v2. Nonetheless, this difference in the SG SF-12v2 scores, as compared to the CG, is mitigated by their minimal difference from the age-adjusted norms (Table 3). Moreover, Lefaivre et al. found that the MPS and IPS have strong construct validity based on correlation with the PCS of the SF-36 [24]. These authors found the weakness of the MPS and IPS to lie in their comparison with the MCS of the SF-36. In this regard, both scores demonstrated ceiling effects (the crowding of the scores at the upper end of the scale, limiting the ability to demonstrate differences between patients with supposedly better clinical outcomes) [24]. Therefore, our data could be interpreted as indicating that for patients with ostensibly the best clinically apparent physical result, both the MPS and the IPS demonstrate a ceiling effect. However, at this high level of physical function, a ceiling effect becomes clinically irrelevant.

## Conclusions

The results of this pilot study indicate that despite having subclinical alterations in gait, asymptomatic pelvic ring injured patients show minimal, if any, evidence of impaired functional outcome following successful reduction of a posterior pelvic disruption treated by CRPSF. Additional study with larger patient numbers and matched-pair design, perhaps using more sophisticated gait analysis methodology, would be required to further characterize the importance of these abnormalities.
